# Electrochemical Droplet Sculpturing of Short Carbon Fiber Nanotip Electrodes for Neurotransmitter Detection

**DOI:** 10.1021/acselectrochem.5c00135

**Published:** 2025-06-16

**Authors:** Yuanmo Wang, Pankaj Gupta, Ajay Pradhan, Raphaël Trouillon, Jörg Hanrieder, Henrik Zetterberg, Ann-Sofie Cans

**Affiliations:** † Department of Chemistry and Chemical Engineering, Chalmers University of Technology, Kemigården 4, Gothenburg 412 96, Sweden; ‡ Department of Psychiatry and Neurochemistry, Institute of Neuroscience & Physiology, 3570 The Sahlgrenska Academy at the University of Gothenburg, Mölndal 431 80, Sweden; § Department of Electrical Engineering, 5596 Polytechnique Montréal, Montréal H3T 1J4, Canada; ◆ TransMedTech Institute, Montréal H3T 1J4, Canada; ○ SNC Research Group, Montréal H3T 1J4, Canada; ∥ Department of Neurodegenerative Disease, UCL Institute of Neurology, Queen Square, London WC1N 3BG, United Kingdom; ⊥ Clinical Neurochemistry Laboratory, Sahlgrenska University Hospital, Mölndal 431 80, Sweden; # UK Dementia Research Institute at UCL, London WC1N 3BG, United Kingdom; ∇ Hong Kong Center for Neurodegenerative Diseases, Clear Water Bay, Hong Kong 999077, China; ☆ Wisconsin Alzheimer’s Disease Research Center, University of Wisconsin School of Medicine and Public Health, University of Wisconsin-Madison, Madison, Wisconsin 53726, United States

**Keywords:** Carbon fiber nanotip electrode (CFNE), electrochemical etching, microscopy, needle-shaped CFNE, cone-shaped CFNE, electroanalysis, dopamine-loaded liposome

## Abstract

Carbon fiber nanotip electrodes (CFNEs) are crucial for electrochemical recordings of neurotransmission release in confined spaces, such as synapses and intracellular measurements. However, fabricating CFNEs with small surface area to minimize noise remains challenging due to inconsistent tip size control, low reproducibility, and low fabrication success rate. Here, we present a reliable, user-friendly method with high reproducibility and success rate for precise CFNE fabrication using microscopy-guided electrochemical etching of cylindrical carbon fiber microelectrodes in a potassium hydroxide droplet. The electrode positioning at the droplet’s liquid–air interface determines the etched region, while manually applied time- and amplitude-controlled voltage pulses regulate material removal. Hence, real-time adjustments to electrode positioning and incremental voltage pulses enable precise sculpturing, akin to woodcarving with a knife. Using this method, we demonstrate successful fabrication of short (10 μm) CFNEs with tip diameters of 100 nm, with excellent electrochemical properties and sculptured into cone- and needle-shaped electrodes. Employing these CFNEs for low-noise amperometric dopamine (DA) detection from individual 200 nm DA-loaded liposomes, combined with *in silico* simulations, revealed that electrode shape influences detection efficiency based on vesicle size. These findings highlight the critical role of electrode geometry in vesicle-based electroanalysis.

## Graphical abstract

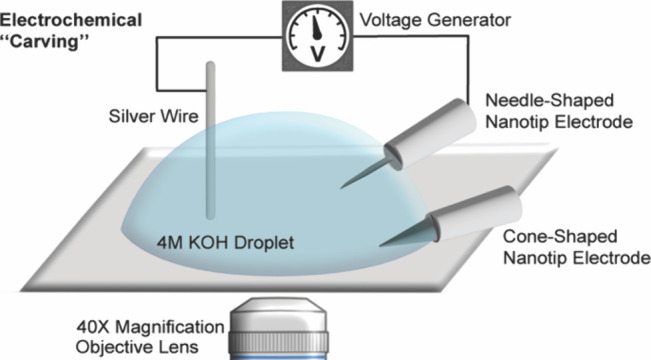

## Introduction

In electrochemical recordings of neurotransmission, selecting the appropriate electrode is crucial, as the electrode material and size must align with the biological environment, where measurements are conducted. Carbon fiber microelectrodes (CFMEs) are particularly favored for detection of neurotransmitters due to their excellent conductivity, biocompatibility, resistance to biofouling, and ability to adsorb numerous electroactive neurotransmitters.^1–4^ Most importantly, they provide stable baselines and enable sub-millisecond resolution of exocytotic events in amperometric recordings.

For *in vivo* brain measurements, CFMEs with diameters of 10–30 μm are small enough to be inserted with minimal tissue damage.^1,5,6^ *In vitro* studies on single cells typically use electrodes of microns in diameter^5–7^ for placement on cell soma as small as 6 to 8 μm.^8^ To record from even smaller spaces, such as at individual neuronal synapses or within cells, sharp carbon fiber nanotip electrodes (CFNEs) with tip diameters around 100 nm are often used.^6,9^

Common fabrication methods for CFNEs, including flame^9–11^ and electrochemical etching,^12^ often lack precision and reproducibility. These techniques, which involve manual handling and uncontrolled exposure of carbon fibers to heat or etchants, offer limited real-time control and frequent damage of the glass seal, resulting in low fabrication success rates and variable electrode geometries. Moreover, these methods often produce excessively long CFNEs (50–200 μm), resulting in a larger electrode surface area and thereby lowering signal-to-noise ratios. To mitigate this, post-fabrication insulation using a resistive film (e.g., epoxy resin or phenolic solution) is typically required but introduces its own challenges, such as inconsistent coverage and poor reproducibility.^10,12^

Efforts to improve fabrication, such as advanced nano 3D-printing combined with atomic layer deposition and focused ion beam cutting, have shown promise but require highly specialized equipment, limiting accessibility and scalability.^13^ Therefore, a more practical, controllable, and reproducible fabrication method is urgently needed.

To address these limitations, we developed a voltage-pulse-controlled electrochemical etching technique for CFNEs, which offers precise, real-time control under optical microscopy. By submerging the bare carbon surface of a traditional glass-sealed cylindrical CFME into a potassium hydroxide (KOH) droplet at the liquid–air interface, ensuring that only the portion of the tip intended for etching is submerged into the droplet while the rest of the electrode remains exposed to the air, we selectively etch the exposed carbon segment while preserving the rest of the structure. This method eliminates the need for post-etching insulation and enables fine control over the tip geometry and length, producing shorter electrodes with smaller surface areas that are highly sensitive and nanotip electrodes with tip diameters of 100 nm or less.

Here, we demonstrated the fabrication of CFNEs with both needle- and cone-shaped geometries suitable for extracellular synaptic and intracellular measurements. These electrodes were tested using dopamine (DA)-loaded liposomes, where sub-millisecond picoampere amperometric spikes confirmed successful detection of single-vesicle DA release. The transients were attributed to the oxidation of DA released from individual liposome ruptures in response to the electrode potential. The distinct electrode geometries showed a selection for liposomes based on size, offering insights into the interaction between the vesicles and the CFNE surface during electroanalysis. Overall, our etching method provides a reproducible and accessible route for producing high-performance CFNEs with tailor made geometries for advanced electrochemical applications with broad utility across neuroscience, cell biology, and bioanalytical chemistry.

## Materials and Methods

### Fabrication of Carbon Fiber Microelectrodes (CFMEs)

5 μm cylindrical glass-insulated carbon fiber microelectrodes with a 50 μm long extruding bare carbon fiber were fabricated using previously described methods.^14^ Briefly, individual carbon fibers, 5 μm in diameter, were aspirated into borosilicate glass capillaries (1.2 mm O.D., 0.69 mm I.D., Sutter Instrument Co., CA, USA) using a vacuum-assisted system consisting of a vacuum pump (LABOPORT, KNF Neuberger, Inc., NJ, USA), rubber tubing, a 3-way distilling adaptor, and a rubber dropper bulb. The capillaries containing carbon fibers were then pulled using a micropipet puller (Model P-1000, Sutter Instrument Co., CA, USA) with optimized parameters, producing two tapered glass capillary pieces and creating tight glass-carbon fiber seals around the carbon fiber protruding from the pull site. The protruding carbon fiber was trimmed using a single edge razor blade, leaving approximately 50 μm of fiber beyond the glass junction. The glass capillary-carbon junction was sealed by dipping the tapered side into epoxy resin (EpoTek 301, Epoxy Technology, MA, USA) for 3 min, followed by a 15 s acetone dip to remove excess epoxy. Electrodes were then cured overnight at 100 °C in a drying oven (VENTICELL, MMM Medcenter Einrichtungen GmbH, Germany). These electrodes served as the starting material for all electrochemical etching procedures.

### Experimental Setup for Electrochemical Droplet-Based Etching

To develop a reproducible and straightforward method for fabricating low-noise CFNEs with defined tip geometries, an experimental setup was designed that combined inverted bright-field microscopy imaging with electrochemical etching tools. As shown in Figure 1, the CFNE fabrication was conducted on the stage of an inverted microscope (Leica DM IRB, Leica Camera AG, Germany) by inserting the CFME tip into a 30 μL droplet of 4 M KOH placed on a glass microscope coverslip positioned on a microscope stage. The CFME tip at the liquid-air interface was visualized under 40× magnification and projected onto a computer screen via a microscope-linked GigE Vision camera (Manta G-235B ASG, ALLIED Vision Technologies GmbH, Germany). With the aid of a standard hemocytometer, a calibrated ruler was placed on the computer screen, providing real-time assessment of electrode dimension throughout the etching process. A micromanipulator (NARISHIGE Group, Japan) and stage control knobs were used to precisely adjust the electrode’s position within the KOH droplet during etching. This ensured that only the electrode material intended for etching remained inside the KOH droplet, allowing precise control over the carbon fiber etching. Importantly, the electrode was inserted into the droplet at a shallow angle rather than horizontally. This orientation ensured that the glass–carbon interface remained approximately parallel to the curved surface of the KOH droplet, promoting uniform radial etching. It also enabled clear microscopic visualization of the tip throughout etching and avoided mechanical interference from the larger electrode holder, which would otherwise contact the stage before the carbon fiber could reach a fully horizontal position. A voltage pulse generator (DS2A - Mk.II model, Digitimer Ltd., UK) was used to apply time- and amplitude-controlled voltage to the CFME surface versus a silver electrode. This stepwise approach using smaller increments of applied voltage pulses prevented excessive carbon etching and selectively removed the carbon fiber submerged in the KOH, allowing for fine control over electrode shape and length.

**Figure 1:**
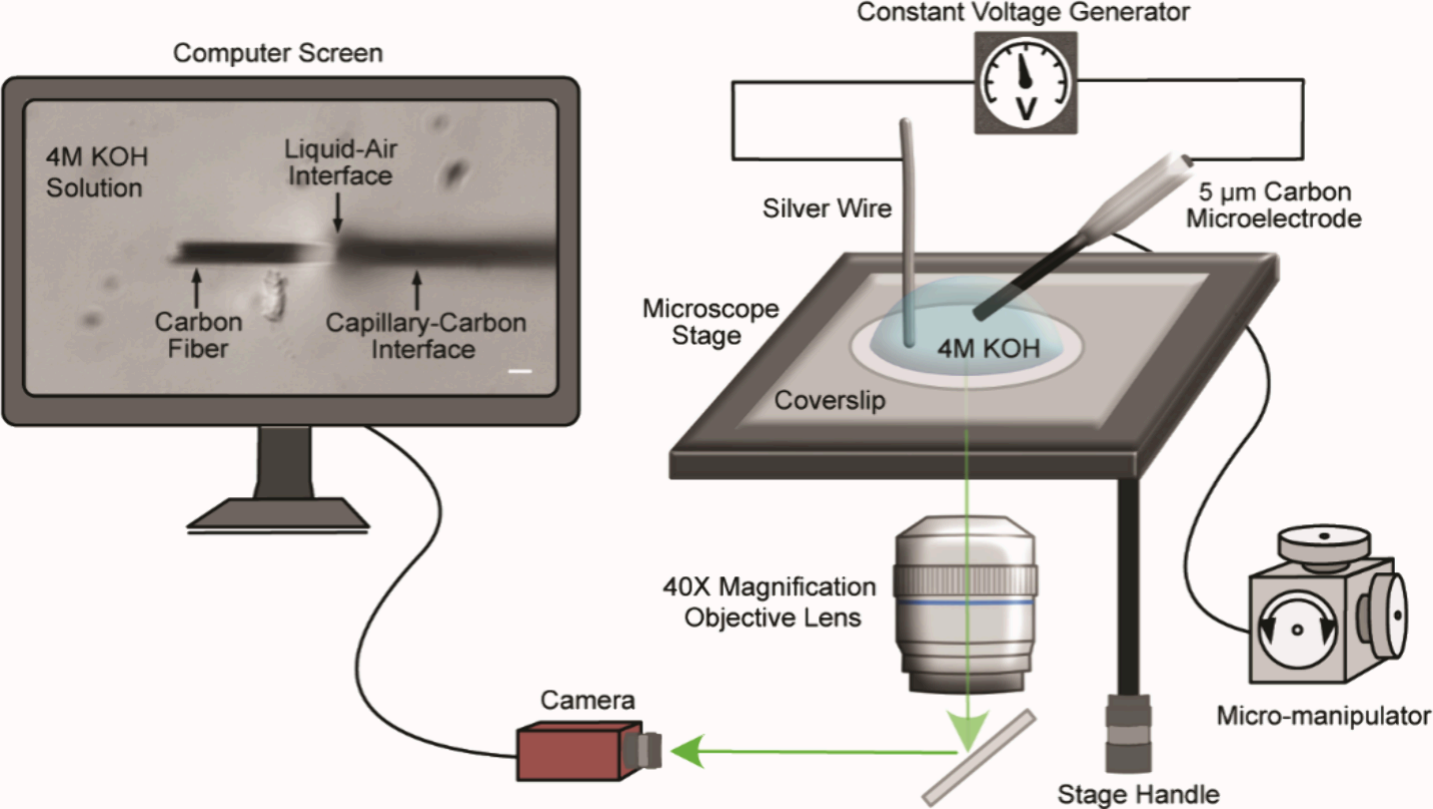
Experimental setup for gentle, stepwise electrochemical etching of carbon fiber nanotip electrodes (CFNEs) with real-time microscopy imaging and camera projection onto a computer screen. A standard hemocytometer was used to create a calibrated ruler on the computer screen. The movable microscope stage and a micromanipulator are used to adjust the placement of the carbon fiber microelectrode (CFME) tips at the liquid-air interface of a 4 M potassium hydroxide (KOH) droplet serving as the etching solution. A voltage pulse generator (Digitimer DS2A-Mk.II) was used to apply time- and amplitude-controlled voltage pulses to the electrode surface versus a silver electrode. The white scale bar on the computer screen (left) is 5 μm. Schematics are not drawn to scale.

### Fabricating Needle-Shaped CFNEs

To produce needle-shaped CFNEs, the entire 50 μm protruding carbon fiber of the CFME was immersed into the 4 M KOH solution near the center of the droplet without submerging the glass-carbon junction (Figure 3A). Initially, 200 ms voltage pulses of 4 V were applied, narrowing the original fiber diameter from 5 μm to ∼ 2.5 μm. Subsequent etching with 3 V pulses (200 ms) was used to further reduce the diameter to ∼1 μm and shorten the fiber length to ∼25 μm. To further gently remove excess carbon at the electrode tip, final fine-tuning was performed using 2–2.5 V pulses, reducing the carbon fiber size below 1 μm and the final length to ∼10 μm. To ensure the preservation of the carbon tip’s integrity within the insulating glass, the glass capillary-carbon junction was kept close but above the liquid surface throughout the etching process.

### Fabricating Cone-Shaped CFNEs

Cone-shaped CFNEs were fabricated by immersing ∼30 μm of the cylindrical CFME tip into a 4 M KOH droplet placed on a glass microscope coverslip (Figure 3B). Multiple 200 ms pulses of 4 V (vs a silver wire) were applied to fully etch the immersed portion and form an initial ∼30 μm long cone-shaped nanotip prototype. Subsequently, ∼20 μm of the remaining carbon fiber was inserted into the KOH droplet so that ∼10 μm remained outside. An additional 200 ms pulses at 3 V shortened the fiber to ∼20 μm. Final shaping with 2–2.5 V pulses refined the cone-shaped nanotip to ∼10 μm in length while avoiding etching near the glass capillary-carbon boundary. The final stage of etching was conducted near the coverslip-liquid interface with careful positioning to ensure sufficient carbon fiber remained outside the liquid.

### Testing CFNE Damage

This method also offers a distinct advantage by enabling real-time detection of electrode damage during fabrication. In conjunction with CFNE droplet etching, electrode integrity was verified by immersing the glass-sealed portion of each electrode into the KOH droplet and applying a potential of 2.0 V (vs a silver wire) for 200 ms; flaws in the glass seal can be quickly identified. The emergence of gas bubbles at the glass/electrode interface was used as an indicator of compromised insulation. Such flaws typically lead to elevated background noise, and therefore, electrodes were deemed unsuitable for use, ensuring that only electrodes of the highest quality proceed for use in experiments.

### Characterization of CFNEs Using Cyclic Voltammetry

All etched CFNEs were characterized by using cyclic voltammetry (CV) in a solution of 1 mM ferrocenemethanol (FcMeOH). Scans from −0.2 V to +0.8 V versus a saturated Ag/AgCl reference electrode were performed at a scan rate of 0.1 Vs^–1^ using a multi-channel potentiostat (1030C, CH Instruments, TX, USA). Electrodes were selected for further use only if they showed stable and characteristic voltammograms.

### Estimation of CFNE Dimensions from SEM Images

High-resolution imaging of etched CFNEs was conducted using a JEOL JSM-7800F Prime Field Emission Scanning Electron Microscope (JEOL GmbH, Germany). Electrodes were backfilled with silver paste and fitted with a silver wire for the electrical connection. The silver paste was allowed to dry completely to ensure a secure and reliable electrical connection. For high resolution, stable imaging, the CFNEs were firmly grounded to the scanning electron microscopy sample stage by using a combination of carbon tape and copper tape.

To characterize the dimensions of two CFNE types (electrode length, tip size, and width), the SEM images were analyzed in ImageJ software (NIH). For estimation of the electrode total surface area, calculations were performed using the following geometric equations for cone-shaped CFNEs (*A*_cone_) and for needle-shaped CFNEs (*A*_needle_):

1 $$A_{cone}=\pi \frac{d_{base}}{2}\left(\frac{d_{base}}{2}+\sqrt{h^{2}+{\left(\frac{d_{base}}{2}\right)}^{2}}\right)$$

2 $$A_{needle}=A_{cylinder}+A_{base}=2\pi \left(\frac{d_{body}}{2}\right)h+\pi {\left(2.5\right)}^{2}$$

### Preparation of DA-Loaded Liposome

Dopamine (DA)-loaded liposomes were synthesized using a lipid mixture of DOPC, DOPE, and cholesterol (39:21:40 molar ratio, Avanti Polar Lipids, Inc., AL, USA) in 3 mL of chloroform. Lipids were dried in a round-bottom flask using a rotary evaporator (Rotavapor R-114, BUCHI Labortechnik GmbH, Germany) at 40 °C for 3 h to ensure evaporation of all organic solvent.^15^ The film was rehydrated for 30 min at room temperature in 200 mM DA dissolved in 10 mM HEPES buffer (pH 7.4, 359 mOsm/kg) to a final lipid concentration of ∼1.5 mg/mL. To ensure complete and even encapsulation of DA solution, the liposomes were subjected to five freeze-thaw cycles, alternating between immersion of the liposome sample vial in liquid nitrogen and a room-temperature water bath.^16^ To unify the liposome size, liposomes were extruded 21 times through a 200 nm pore size polycarbonate membrane (Whatman, UK) using a Mini-Extruder (Avanti Polar Lipids, Inc., AL, USA) under 1 bar of nitrogen gas pressure. Non-encapsulated DA was removed using illustra Microspin S-200 HR columns (GE Healthcare, UK). All steps were performed under nitrogen gas to prevent DA oxidation. An isotonic HEPES-buffered solution (355 mOsm/kg) was prepared by dissolving NaCl salt in 10 mM HEPES (pH 7.4) for use in amperometric recordings.

### Liposome Size Measurement via Nanoparticle Tracking Analysis

Liposome size was measured via a nanoparticle tracking analysis (NTA) system using a NanoSight LM10 (Malvern Instruments Ltd, UK). Liposome samples were diluted 2000 times in isotonic HEPES buffer after removal of non-encapsulated DA. Further dilution was made as needed to achieve optimal NTA particle concentration. Measurements were conducted at room temperature with five 1 min runs and three measurements for each freshly prepared liposome sample.

### Amperometric Detection of DA Released from Liposomes

Prior to measurement, CFNEs and a chloride silver wire reference electrode were immersed in the liposome sample solution, diluted 1-5 times in isotonic HEPES buffer. Only CFNEs that exhibited a stable performance in cyclic voltammetry were used. A constant potential of +700 mV (versus chloride silver wire) was applied to the CFNEs using an Axopatch 200B patch clamp amplifier and an Axon Digidata1550B digitizer (Molecular Devices, CA, USA) for a 3–5 min amperometric recording. Liposomes near the electrode surface are destabilized by the local electronic field and subsequently rupture upon contact with the electrode surface, releasing their DA content for electrochemical detection, where single vesicle DA release is recorded as discrete amperometric spikes. Data were digitized at 20 kHz with a 1 kHz low-pass Bessel filter.

### Amperometric Data Analysis

Spike analysis was conducted using an open-source script from David Sulzer’s lab,^17^ implemented in Igor Pro 6 software (WaveMetrics, OR, USA). The raw amperometric data were smoothed with a 5 kHz binomial filter. Spikes exceeding 5 times the standard deviation of the background noise were classified as DA release events. All amperometric data were manually inspected, and false positive spikes were removed. Only recordings with >60 spikes were analyzed. Extracted parameters enabling detailed analysis and characterization of the amperometric transients included: peak current amplitude (*I*_max_), rise time (*T*_rise_), and fall time (*T*_fall_) representing the time between 25% and 75% of *I*_max_, the total spike time (*T*_base_), spike-half-time (*T*_1/2_) denoted the spike half-width at 50% of *I*_max_, and the total charge (*Q*), which represents the total charge detected from the content release during individual liposome rupture. From the measured *Q*, the number of DA molecules released per vesicle (*N*) can be quantified using Faraday’s law: *N* = *Q*/*nF*, where *n* is the number of electrons transferred in each oxidation reaction (*n* = 2 for DA) and *F* is the Faraday’s constant (96,485 C/mol). Hence, the recorded *Q* was used here to determine the number of DA molecules released per liposome and compared to the detected DA content released from individual liposomes using these two types of CFNEs.

### Amperometric Current Simulations

Simulations were conducted using COMSOL Multiphysics 6.1 (COMSOL Inc., Sweden) with the Transport of Diluted Species module. The aqueous model domain was a cylinder (2 μm in diameter and 1 μm height) with free analyte diffusion. The electrode was represented as a sphere (diameter = *D*_elec_) and the liposome, as another sphere (diameter = *D*_lip_), containing 200 mM DA (diffusion coefficient of 6 × 10^–10^ m^2^ s^–1^) with an impermeable surface except for a pore (diameter = *D*_pore_). To simulate diffusion-limited oxidation of DA, the analyte concentration was set to 0 mM at the electrode surface. Electrode-pore distances of 1, 5, and 20 nm were simulated. To accommodate the small scale of the system, a tetrahedral mesh (0.0–80 nm size) was applied, and simulations were run over 10 ms in 0.01 ms intervals. Current was calculated from the normal analyte flux across the electrode surface using Faraday’s law.

## Results and Discussion

### Optimizing Protocols for Electrochemical Droplet Carving of CFNEs

To establish a robust and time-efficient method for creating short CFNEs with defined geometries, we designed an experimental setup that integrates inverted bright-field microscopy with electrochemical etching and optimized a gentle, stepwise electrochemical droplet-based etching protocol (Figure 1), allowing precise control over the carbon fiber etching, similar to wood sculpting with a knife. Using this setup, experimental conditions were optimized to create two distinct electrode tip geometries: needle- and cone-shaped CFNEs. Since the duration and voltage applied to the electrode surface directly influence both the extent and rate of carbon fiber removal, we systematically explored these variables. The voltage generator allowed for temporal variation from 20 μs to 2 s and voltage amplitudes between 0 and 99 V. In these protocols, voltage amplitudes (3–4 V) and pulse durations (200 ms to 2 s) were systematically evaluated to remove material from the 50 μm long bare carbon fiber protruding from the 5 μm CFME base when immersed into a 4 M KOH droplet, serving as the electrochemical etching solution.

In the initial stage, a series of high-voltage pulses (∼4 V) were used to quickly carve away the bulky portion of the 50 μm long carbon fiber extending from the glass-sealed 5 μm cylindrical CFME (Stage I, Figure 2A) submerged in the KOH droplet. To assess efficiency, the etching time required to produce a 30 μm long cone-shaped CFNE (Stage II, Figure 2B) electrode was first systematically minimized by evaluating voltage amplitudes of 3 and 4 V and pulse durations of 200 ms, 1 s, and 2 s (Figure S1). Etching time was monitored in real-time using on-screen imaging and a calibrated ruler. The voltage pulses were manually repeated until the carbon fiber tip was visibly carved into its final cone shape. The number of manually applied voltage pulses needed to visibly form the final cone shape of the electrode (stage II, Figure 2B) was counted. Importantly, while the voltage and pulse durations were predefined for each etching stage, the number of pulses was dynamically adjusted in real time based on visual feedback from microscope imaging, allowing the operator to terminate etching upon reaching the desired electrode shape and length. Figure 2C,D shows that 3 V with 200 ms pulses was the most efficient and 4 V further reduced etching time, leading us to select 200 ms pulses throughout the study, and for more delicate shaping of the electrode tip, lower amplitudes were applied.

**Figure 2:**
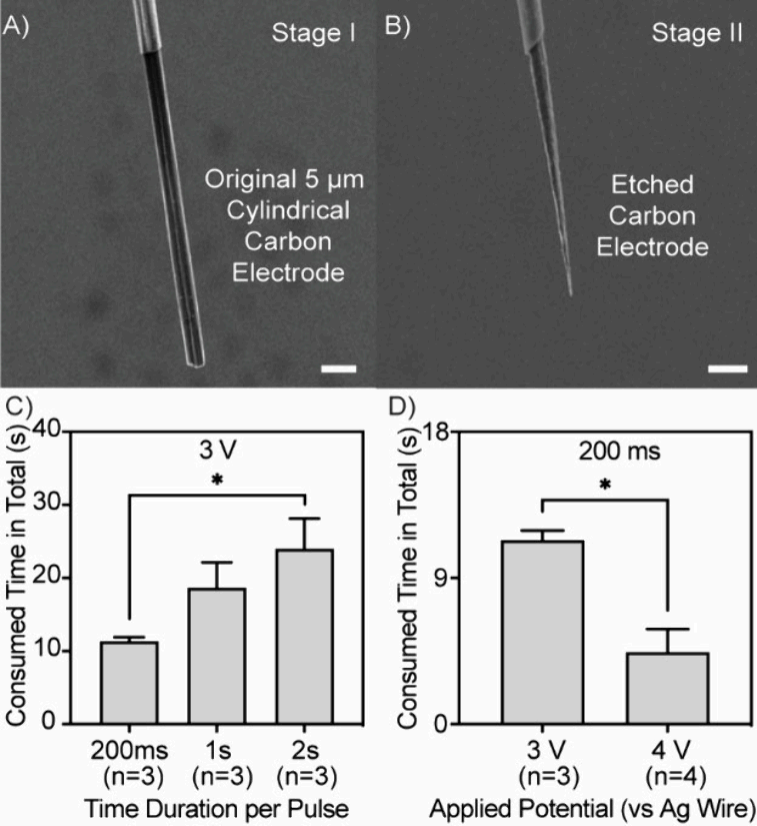
Scanning electron microscopy images of (A) a representative 50 μm long, 5 μm cylindrical carbon fiber microelectrode (CFME) before etching (Stage I) and (B) a 30 μm long cone-shaped carbon fiber nanotip electrode (CFNE) (Stage II) produced after KOH droplet etching. Scale bars in parts (A) and (B) represent 10 μm. (C, D) Analysis of etching efficiency and total etching time during the etching of a cylindrical CFME into a cone-shaped nanotip electrode. In (C), etching time was evaluated by varying pulse durations (200 ms, 1 s, and 2 s) while applying 3 V pulses. In (D), etching time was assessed by varying the applied potential (3 and 4 V) while keeping the pulse duration constant at 200 ms. Data are presented as the mean ± the standard error of the mean (SEM). A two-tailed unpaired *t*-test was performed for comparison, *p < 0.05.

### Optimization of KOH Concentration for Droplet Etching

KOH concentration plays a critical role in determining both the kinetics of carbon etching and the resulting surface properties, such as roughness, porosity, and chemical functionality.^18^ To establish optimal etching conditions for fabricating high-aspect-ratio CFNEs, we systematically evaluated the effect of the KOH concentration through an empirical, trial-and-error approach.

In our experiments, lower concentrations of KOH (e.g., 1 M) resulted in significantly slower etching rates, making it extremely difficult to reproducibly generate sharp conical- or needle-shaped nanotip geometries. This observation is consistent with findings by Venton et al., who reported that applying a constant potential of 1.5 V in 1 M KOH for 3 h resulted in only ∼45% reduction in carbon fiber diameter, which is insufficient for forming the nanoscale tips required for high-sensitivity electrochemical detection.^18^

Our goal of this study was to fabricate CFNEs with sharp tips and short active lengths, which necessitated a faster and more aggressive etching process. Increasing the KOH concentration to 4 M significantly enhanced etching efficiency and enabled the reproducible formation of well-defined cone- and needle-shaped tips within a practical timeframe. We also found that higher concentrations produced smoother tip surfaces, whereas prolonged etching at low KOH concentrations yielded rougher surfaces without achieving the desired geometry.

Supporting this, in a parallel study, we demonstrated that short etching durations in 4 M KOH result in smooth carbon surfaces with minimal chemical modification, preserving the electrochemical integrity of the electrode.^19^ Based on these results and the use of 4 M KOH previously reported by Sombers’ group for electrochemical etching,^12^ we selected 4 M KOH as the optimal concentration for droplet etching in this study, providing a balance between etching speed, surface quality, and tip geometry reproducibility.

### Controlling Electrode Placement in the KOH Droplet

To further refine electrode geometry and length, the electrode position within the KOH droplet was optimized (Figure 3). This included optimizing both the depth of the electrode tip submerged into the KOH droplet and its insertion angle with respect to the curved liquid interface from the top to the bottom of the KOH droplet for creating the two different needle- and cone-shaped short CFNEs, as illustrated by the schematic in Figure 3A,B from stages I–IV. During the initial high-voltage carving, the CFME was held center-high in the droplet. Subsequently, fine etching involved lowering the electrode toward the coverslip surface and decreasing the applied voltage to 2 V, ensuring gradual shaping with minimal risk of over-etching.

**Figure 3:**
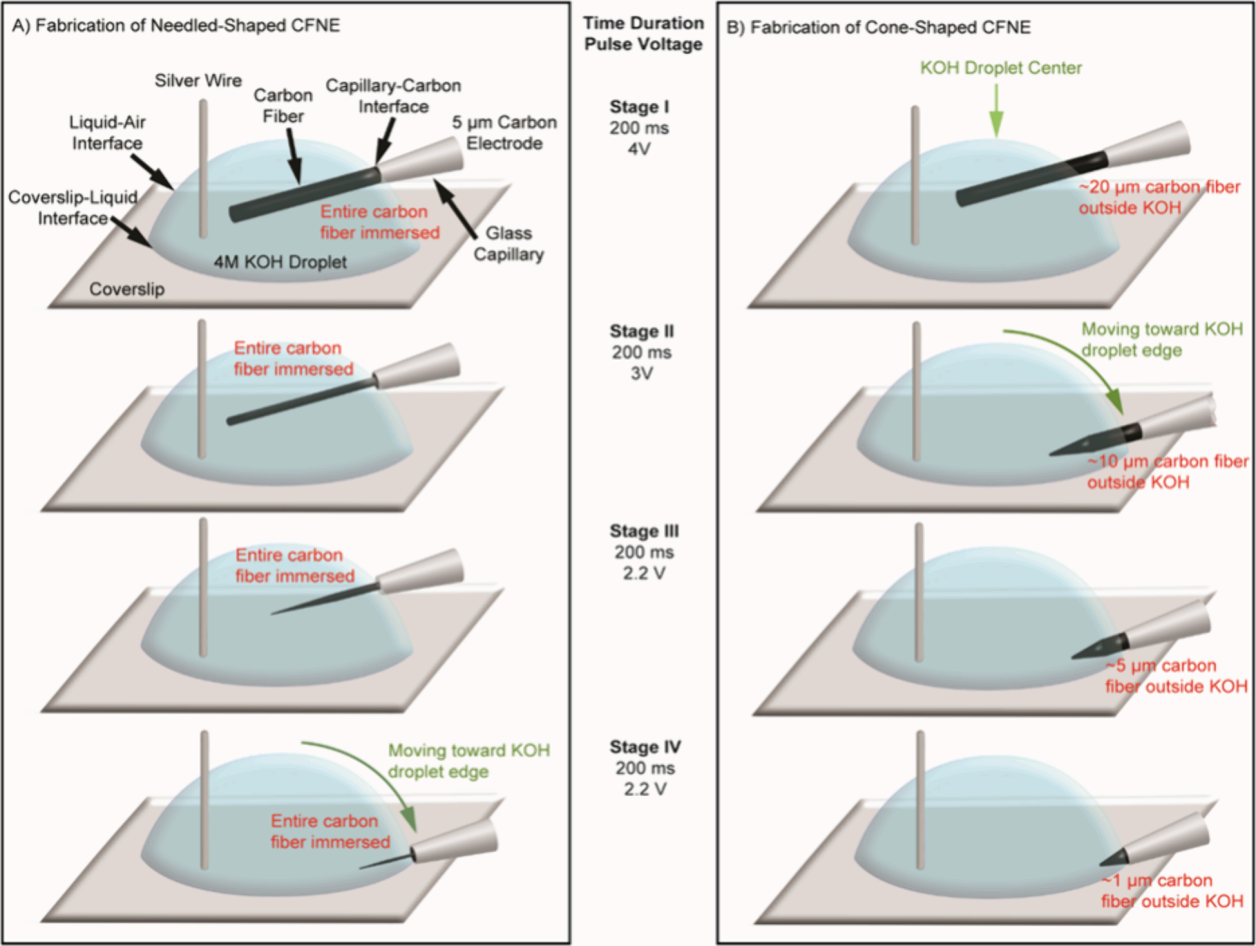
Stepwise electrochemical droplet etching strategy for fabricating CFNEs with distinct geometries. Schematics illustrate the fabrication of (A) needle-shaped (left) and (B) cone-shaped (right) CFNEs using a stepwise etching process. This method relies on applying 200 ms voltage-controlled pulses combined with precise micromanipulation of the electrode position and depth of carbon fiber immersed in a KOH droplet. These parameters are critical for shaping the final geometry of the nanotip. Initially (Stage I), the CFME is positioned at the center-top of the droplet. During the etching process, the electrode tip is gradually lowered toward the edge of the KOH droplet near the microscope coverslip-liquid interface (Stages II–IV). (A) To create needle-shaped tips, the entire cylindrical carbon fiber is fully submerged into the droplet, aligning the glass capillary-carbon fiber interface with the droplet’s liquid-air surface. (B) In contrast, cone-shaped electrode tips are produced by partially submersing the carbon fiber tip into the droplet while keeping a portion of carbon fiber (20 μm to 1 μm) outside the KOH droplet, thereby selectively etching the exposed portion of the fiber. Schematics are not drawn to scale.

### Fabrication of Needle-Shaped CFNEs

Needle-shaped CFNEs were fabricated by fully immersing the entire 50 μm carbon fiber, extending from the glass taper, into the KOH droplet throughout the etching process (Figure 3A; Supporting Information Video 1). Initial etching at 4 V (Stage I) with the carbon fiber was positioned near the center of the KOH droplet (Stage I, Figure 3A), progressively reducing the electrode diameter from 5 to ∼2.5 μm. Subsequent etching at 3 V further reduced the diameter to ∼1 μm and shortened the carbon fiber to ∼ 25 μm (Stage II). Final shaping at 2 to 2.5 V yielded ∼100 nm tips and lengths of 10 μm or less (Stages III–IV) by moving the electrode tip closer to the surface of the glass coverslip during the etching process. In some cases, the desired nanotip electrode dimensions were already achieved after Stage II.

### Fabrication of Cone-Shaped CFNEs

The more robust cone-shaped CFNEs were fabricated by partially immersing the carbon fiber, leaving ∼20 μm of the fiber outside the KOH droplet during the initial etching stage (Stage I, Figure 3B; Supporting Information Video 2). Voltage pulses at ∼4 V were etched on the submerged portion into a preliminary ∼30 μm long cone shape. The carbon fiber was then brought down closer to the glass coverslip surface for continued etching at 3 V, leaving ∼10 μm of carbon fiber outside the droplet, reducing the fiber length to ∼20 μm (Stage II). Lowering the voltage pulse amplitudes to 2–2.5 V further refined the electrode tip shape, reducing its final length to ∼10 μm and resulting in a cone-shaped tip (Stages III and IV) with a ∼100 nm tip size (Figure 4B). The reported values for the carbon fiber segments kept outside the KOH droplet at each stage (e.g., 20 μm, 10 μm, 5 μm, 1 μm) serve as visual positioning guidelines and can be adjusted based on the desired electrode length and shape. In addition, researchers may modify the protocol by using CFMEs of different starting diameters (e.g., 7 μm, 10 μm, and 30 μm) and re-optimizing voltage and pulse duration as needed for their target geometry. All parameters are adjustable in real time using microscope feedback, allowing this method to be broadly adaptable across different fabrication needs. Throughout the process, careful manipulation was essential to prevent etching damage near the glass-carbon interface.

**Figure 4:**
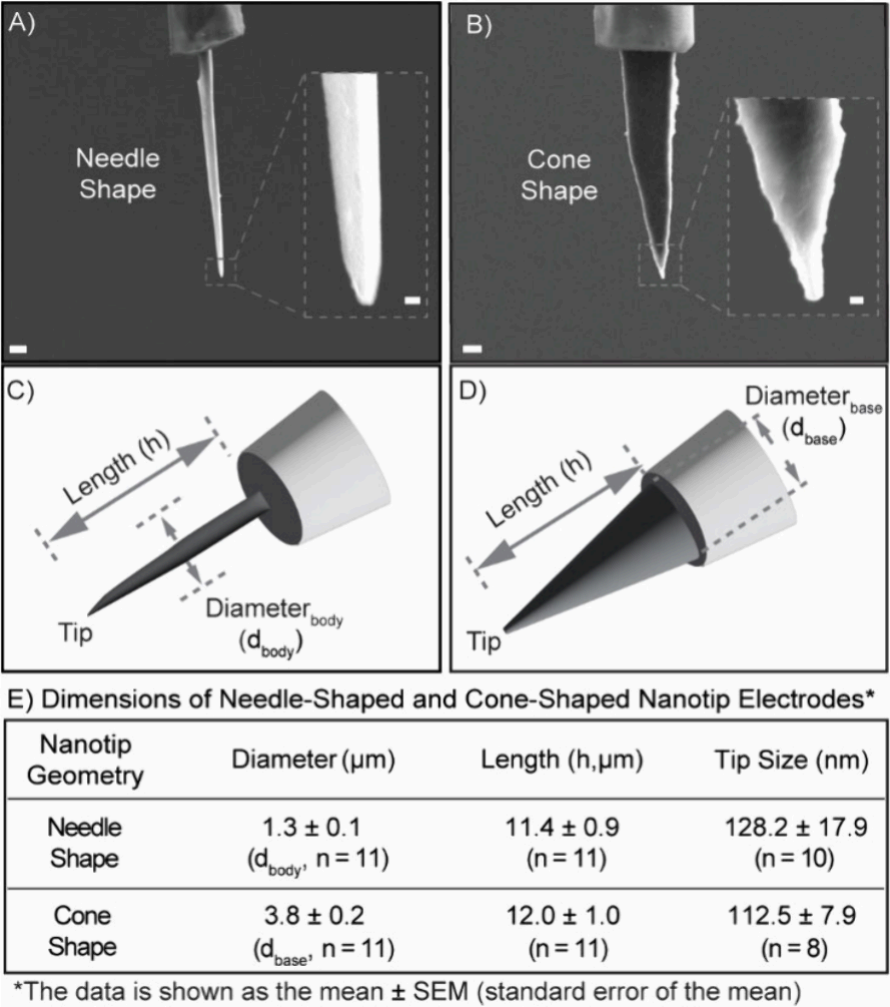
Scanning electron microscopy images of representative (A) needle-shaped and (B) cone-shaped carbon fiber nanotip electrodes (CFNEs) with tip diameters of approximately 100 nm. The scale bars in panels (A) and (B) indicate 1 μm, with additional scale bars of 100 nm shown in the inset images. (C) and (D) illustrate the defined measured dimensions of needle-shaped and cone-shaped CFNEs, respectively. (E) Table summarizing the measured dimensions of needle-shaped and cone-shaped CFNEs as determined by scanning electron microscopy image analysis of the differently shaped CFNEs, reported as mean ± SEM, unless otherwise indicated. While (C) and (D) show idealized axisymmetric schematics to define dimensional parameters, the images in (A) and (B) reflect the physical reality of fabricated CFNEs, which may exhibit minor geometric asymmetries. These result from droplet curvature, variable immersion depth at the droplet edge, and limitations of manual alignment during the fine-tuning of the electrode geometry.

Despite careful alignment, some degree of asymmetry or eccentricity in the tip shape may still occur. This is primarily due to geometric and physical limitations of the KOH droplet interface, particularly during final etching stages, where the carbon fiber is positioned near the edge of the curved droplet. In this region, the liquid depth is shallow and surface curvature may cause uneven etchant contact angles across the fiber surface. Additionally, microscale shifts during manual micromanipulation can lead to small lateral misalignments. These factors together may contribute to deviations from the ideal axisymmetric tip profiles observed in SEM images. Nevertheless, the fabricated CFNEs consistently exhibit controlled tip lengths and diameters (∼10 μm and ∼100 nm, respectively) with excellent electrochemical performance.

### Characterization of CFNE Dimensions

Scanning electron microscopy imaging confirmed distinct geometrical profiles for the two electrode types (Figure 4). Needle-shaped CFNEs were etched into thin, spear-like structures with a relatively uniform thickness from the glass capillary-carbon edge along the electrode base, measuring approximately 1.3 ± 0.1 μm (*n* = 11) to the pointy tip, with tip diameters of 128.2 ± 17.9 nm (*n* = 10) and lengths of 11.4 ± 0.9 μm (*n* = 11). Cone-shaped CFNEs had wider bases (3.8 ± 0.2 μm, *n* = 11), tapering to 112.5 ± 7.9 nm tips (*n* = 8) and lengths of 12.0 ± 1.0 μm (*n* = 11), respectively. The dimension data here was reported as mean ± standard error of the mean (SEM), unless otherwise indicated. Both etching protocols produce short CFNEs with nanoscale electrode tips.

### Characterization of CFNE Electrochemical Performance

To evaluate and compare the electrochemical performance of needle- and cone-shaped CFNEs, we performed cyclic voltammetry (CV) in a 1 mM solution of ferrocenemethanol (FcMeOH) as shown in Figure 5A. Representative voltammograms for both geometries are presented in Figure 5B,C. Both electrode types displayed excellent reaction kinetics with low-noise, stable steady-state currents, which is consistent with intact electrode insulation and small electrode surface areas. These results confirm that the carbon nanotip and glass capillary insulation remained intact after etching. Notably, unlike other studies that require post-etching insulation steps, our fabrication approach eliminates this need by enabling precise real-time visualization and control of the etching process.

**Figure 5:**
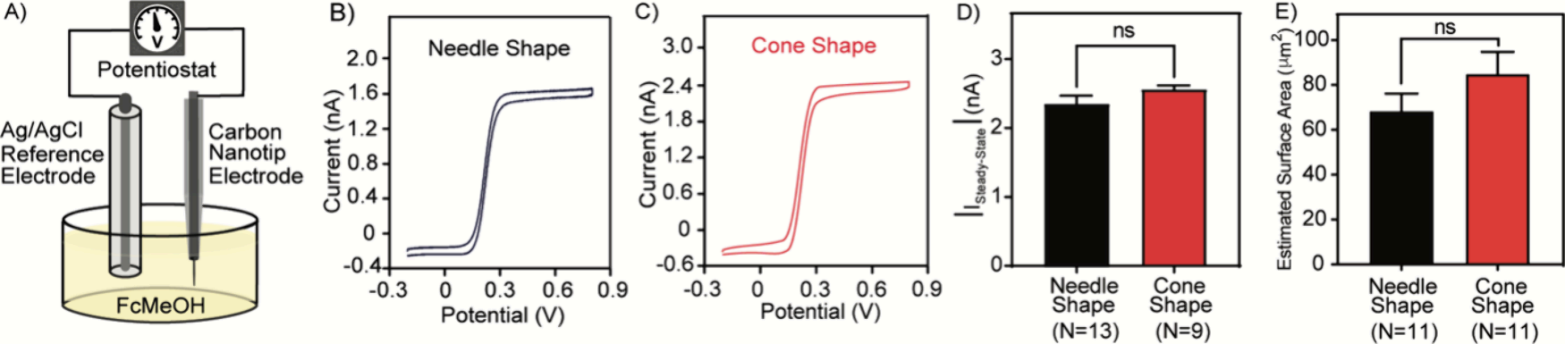
Electrochemical characterization of CFNEs using cyclic voltammetry. (A) Schematic illustration of the experimental setup for cyclic voltammetry (not drawn to scale). Representative cyclic voltammograms of (B) a needle-shaped CFNE (black) and (C) a cone-shaped CFNE (red), recorded in 1 mM ferrocenemethanol (FcMeOH) solution. (D) Comparison of steady-state current amplitude at +0.6 V for needle-shaped (*n* = 13, black) and cone-shaped (n = 9, red) CFNEs. (E) Surface area estimates for needle-shaped (*n* = 11) and cone-shaped (*n* = 11) CFNEs based on SEM analysis. In (D) and (E), data are displayed as the mean ± SEM. The Mann-Whitney (two-tailed, unpaired) test was performed for statistical comparison, with “ns” indicating no significant difference.

To further compare the active surface area and noise level of the two CFNE geometries, we evaluated the steady-state background current amplitude (|*i*_steady‑state_|) generated by the applied potential wave (*v*), which is directly proportional to the electrode surface area and the capacitance, *C*_d_, according to

3 $$\left|i_{steady\text{‐}state}\right|=C_{d}\nu$$

The average steady-state current amplitudes recorded in 1 mM FcMeOH were approximately 2.3 nA (*n* = 13) for needle-shaped CFNEs and 2.6 nA for cone-shaped CFNEs (*n* = 9), with individual values ranging from 1.8 to 2.8 nA (Figure 5D). Statistical analysis revealed no significant difference in current amplitude between the two types, suggesting comparable active carbon surface areas despite differences in geometry. To verify these findings, the electrode surface areas were estimated from the scanning electrode microscopy images of the CFNEs (Figure 4A,B) and electrode dimensions measured using ImageJ software (Figure 4C–F). These estimations (Figure 5E) confirmed no significant difference in the calculated surface area between the two CFNE types. However, it is important to note that the etching process for needle-shaped CFNEs exposes additional carbon surface area at the glass capillary-carbon interface, which contributes to the total active surface area and thereby may influence steady-state current values. Additionally, the steady-state currents recorded from both CFNE types were significantly lower than those measured using conventional 30 μm diameter, 45° angle beveled disc carbon electrodes (Figure S2), a commonly used electrode in quantal neurotransmitter analysis^20,21^ and amperometric measurements in brain tissue slides.^22^ This lower background current reflects the significantly smaller tip surface area of CFNEs and underscores their suitability for low-noise, high resolution electrochemical recordings in single-cell and nanoscale neurochemical applications.

### CFNEs Enable Picoampere-Sensitive Detection of DA Release from Liposomes

Vesicular neurotransmitter content can be quantified using amperometry by applying a constant potential to an electrode surface that is either inserted into the cell cytoplasm or placed in a solution of isolated vesicles, which triggers vesicle rupture upon contact.^9,20,23^ This results in quantal release of neurotransmitter molecules and transient current spikes from oxidizing these molecules upon release. The total integrated charge of a spike reflects the total number of oxidized molecules, while the rise and fall times report on the kinetics of vesicle rupture and release. Accurate resolution of these events, which occur on a sub-millisecond time scale, requires electrodes with high temporal resolution, low baseline noise, and picoampere-level sensitivity, particularly for detecting small synaptic vesicles containing as few 5,000–10,000 molecules typically compared to the larger amounts of 100,000–300,000 molecules found in large dense core vesicles.^24–26,21,14^ To evaluate the performance of the droplet etched CFNEs, we recorded amperometric signals from detecting DA release from small synthetic liposomes designed to mimic neurotransmitter vesicles. DA-loaded liposomes were prepared with a mean diameter of ∼148 nm and a mode diameter of ∼221 nm, as measured by nanoparticle tracking analysis (Figure S3), and the CFNEs were immersed in the liposome solution under constant applied potential (+700 mV potential vs a chloriding Ag wire). Upon collision and spontaneous rupture at the electrode surface, liposomes released their contents, generating characteristic amperometric quantal current spikes (Figure 6A,B). Spikes were recorded at 20 kHz and filtered at 5 kHz to accurately analyze kinetic and quantitative spike parameters including peak amplitude (*I*_max_), rise time (*T*_rise_), fall time (*T*_fall_), base time (*T*_base_), half time (*T*_1/2_), and total integrated charge (*Q*) (Figure 6C). Both needle- and cone-shaped CFNEs demonstrated remarkable ability to detect DA release from individual rupturing liposomes with sub-millisecond temporal resolution and picoampere sensitivity (Table S1). With their enhanced spatial resolution and shortened electrode tip lengths, which minimize the active surface area and reduce baseline noise, the geometric design of CFNEs plays a crucial role in enabling high-sensitivity electrochemical measurements. The CFNEs used in this study allow precise amperometric detection of exocytotic release in highly confined spaces, such as neuronal synapses, and enable the intracellular quantification of neurotransmitter content released from secretory vesicles. Previous studies using 30–100 μm carbon fiber tips reported baseline currents of ∼20 pA,^26^ while disk electrodes with diameters of ∼33 μm produced baseline currents of ∼10 pA during catecholamine vesicle detection.^20^ In contrast, our short (∼10 μm) needle- and cone-shaped CFNEs with ∼100 nm tip diameters consistently exhibit baseline currents of ∼2 pA and ∼5 pA, respectively, thereby improving sensitivity for detecting small-amplitude dopamine release events. Since dopamine is a catecholamine, these comparisons are directly relevant to our study.

**Figure 6:**
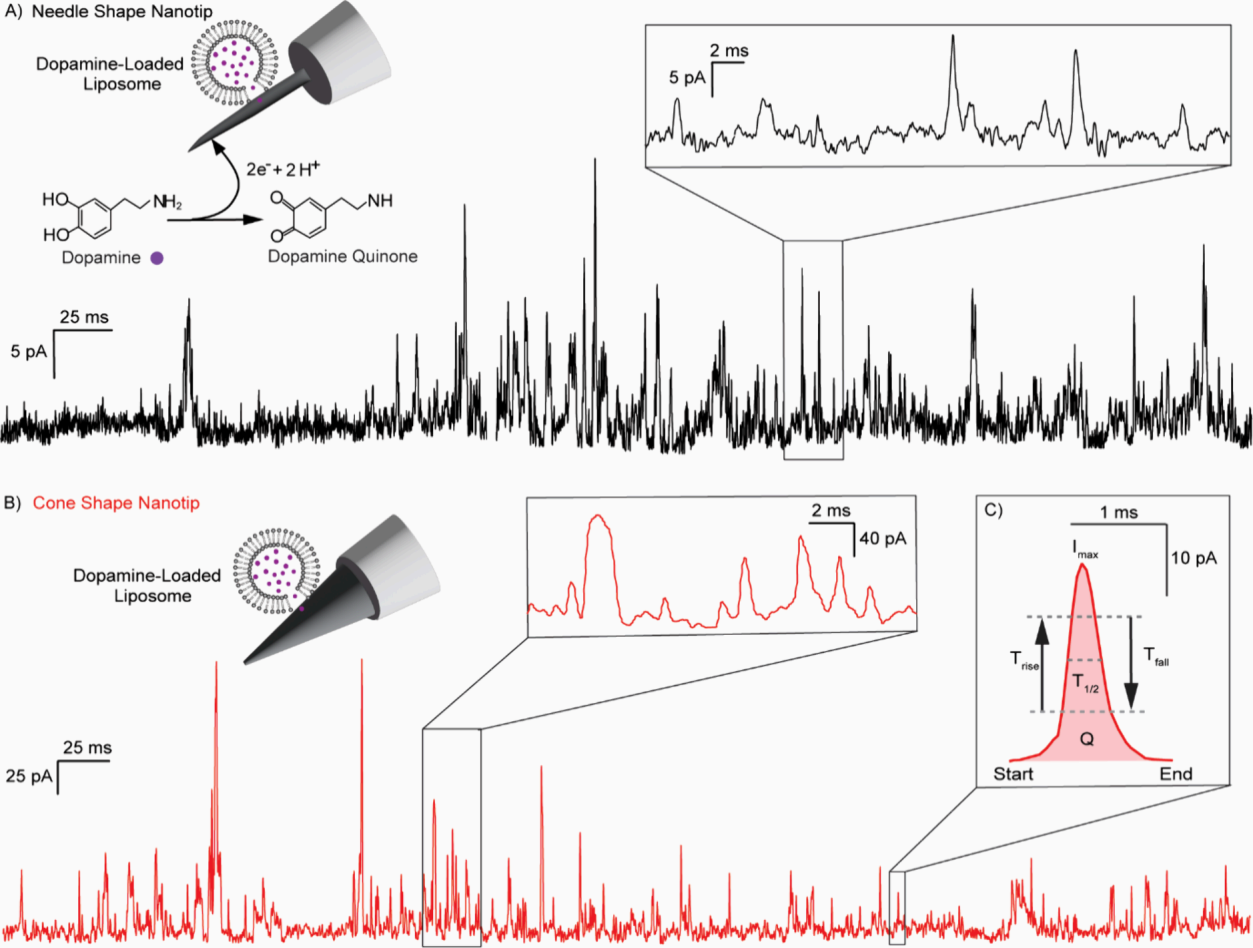
Representative amperometric traces of dopamine (DA) release from individual liposomes ruptured at the surface of CFNEs. Single liposomes containing 200 mM DA stochastically ruptured and detected by (A) needle-shaped (black) and (B) cone-shaped (red) CFNEs, according to the mechanism illustrated in the accompanying schematics (not drawn to scale). (C) Expanded view of a representative current spike showing DA release from a single liposome with labeled kinetic and quantitative parameters used for DA quantification and kinetic analysis.

## Electrode Geometry Influences Detection Sensitivity and Vesicle Size Resolution

### Cone-Shaped CFNEs Offer Superior Signal Resolution and Sensitivity

Both needle-shaped and cone-shaped CFNEs successfully detected DA release events from individual liposomes. However, cone-shaped CFNEs consistently produced larger current spikes, reflected by a significantly larger average spike area (Figure 7A) and amplitude (Figure 7B, Table S1). Using Faraday’s law, we estimated the number of DA molecules released per liposome (Figure 7C). Assuming a Log-normal distribution, cone-shaped CFNEs detected a higher number of DA molecules per liposome (mode ∼35 zmol) compared to needle-shaped CFNEs (mode ∼12.5 zmol). Assuming a 100% detection efficiency from liposomes encapsulating 200 mM DA, this result implies that cone-shaped CFNEs tend to detect larger liposomes (mode inner diameter ∼76 nm), whereas needle-shaped CFNEs detect smaller ones (mode inner diameter ∼52 nm). Despite being recorded from the same liposome preparation, significant differences were observed in the quantitative results obtained with the two CFNE geometries (Figure 7C,D). This discrepancy highlights the importance of understanding how electrode geometry influences detection. We hypothesized that the observed differences arise from the distinct shapes of the CFNEs. Specifically, it is the larger surface area and lower curvature of cone-shaped CFNEs, which may capture a larger fraction of released neurotransmitters, compared to the sharper, more curved needle-shaped CFNEs. To test this hypothesis, we conducted finite element simulations to assess how CFNE geometry affects liposome-electrode interactions and the detection efficiency.

**Figure 7:**
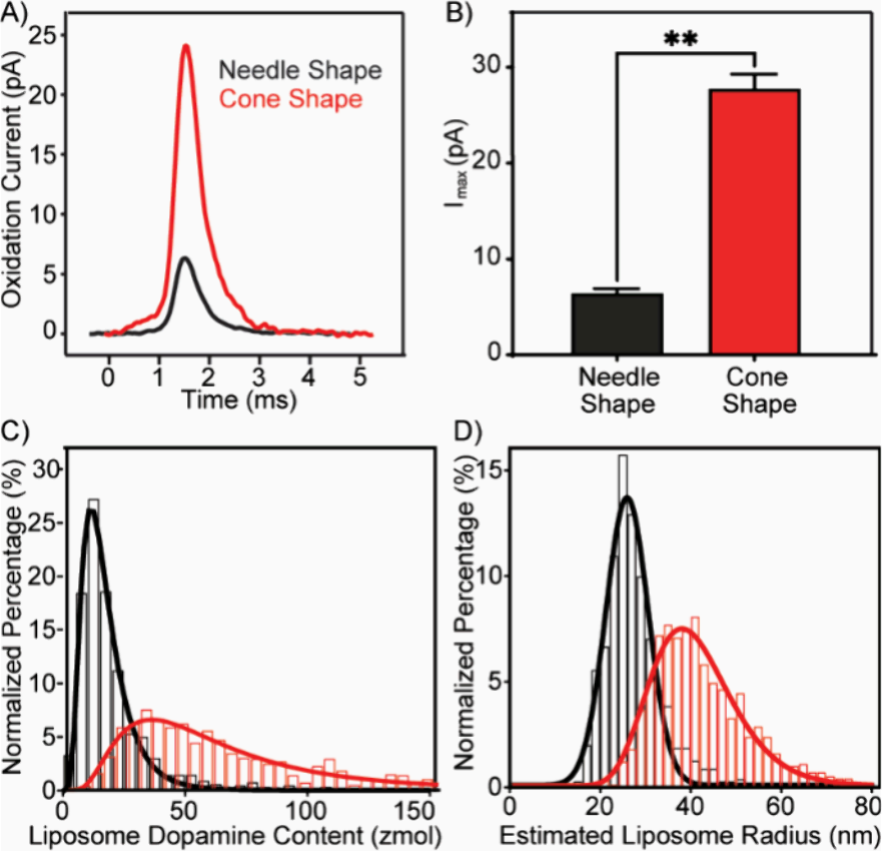
Amperometric analysis of DA release from rupturing liposomes at needle-shaped (black) and cone-shaped (red) CFNEs. (A) Averaged current spikes using needle-shaped (*n* = 356 spikes) and cone-shaped (*n* = 395 spikes) CFNEs. (B) Comparison of current spike amplitudes (*I*_max_) between needle- and cone-shaped CFNEs. Statistical analysis was performed using an unpaired two-tailed *t* test with Welch’s correction (**p < 0.01). (C) Normalized frequency histogram of vesicular DA content, calculated from the recorded charge using Faraday’s law. Data are binned in 5 zmol intervals. Both distributions fit a Log-normal function (*R* ^2^ = 0.99 for needle-shaped CFNEs; *R* ^2^ = 0.97 for cone-shaped CFNEs). (D) Estimated size distribution of rupturing liposomes derived from the detected charge assuming a 200 mM DA concentration. The histogram presents data binned in 2 nm intervals. Log-normal fits yielded a *R* ^2^ of 0.97 for both electrode types. Data in (B–D) represent pooled measurements from 4 needle-shaped CFNEs (*n* = 707 spikes) and 3 cone-shaped CFNEs (*n* = 908 spikes). All values are reported as mean ± SEM unless otherwise stated.

### Finite Element Simulations Reveal Detection Efficiency and Release Dynamics

Amperometric detection primarily relies on the passive mass transport (diffusion) of analytes from the liposome membrane pore to the electrode surface. Therefore, the initial hypothesis was that differences in electrode shape led to distinct diffusion profiles, which could affect detection. The key geometrical parameters under consideration were the electrode-to-liposome size ratio and the surface curvature. Notably, needle-shaped CFNEs exhibit a radius of curvature smaller than that of cone-shaped CFNEs. To investigate these factors, we simulated DA release from 200 nm diameter liposomes containing a 200 mM DA using electrodes of varying diameters (10 nm to 10 μm) (Figure 8A–C, Figure S4A,B). A spherical electrode with a diameter (*D*_elec_) was used in the model to emphasize the effects of the curvature. The liposome membrane pore diameter (*D*_pore_) was set to 4.5 nm, matching experimental current kinetics (*T*_1/2_ ∼ 0.5 ms, Table S1). The liposome-electrode distance (*d*) was set to 1 nm, which is consistent with previous studies.^15^ The simulations showed that, under these conditions, the current spike characteristics were largely independent of *D*_elec_, indicating that local curvature does not explain the quantitative differences observed in Figure 7.

**Figure 8:**
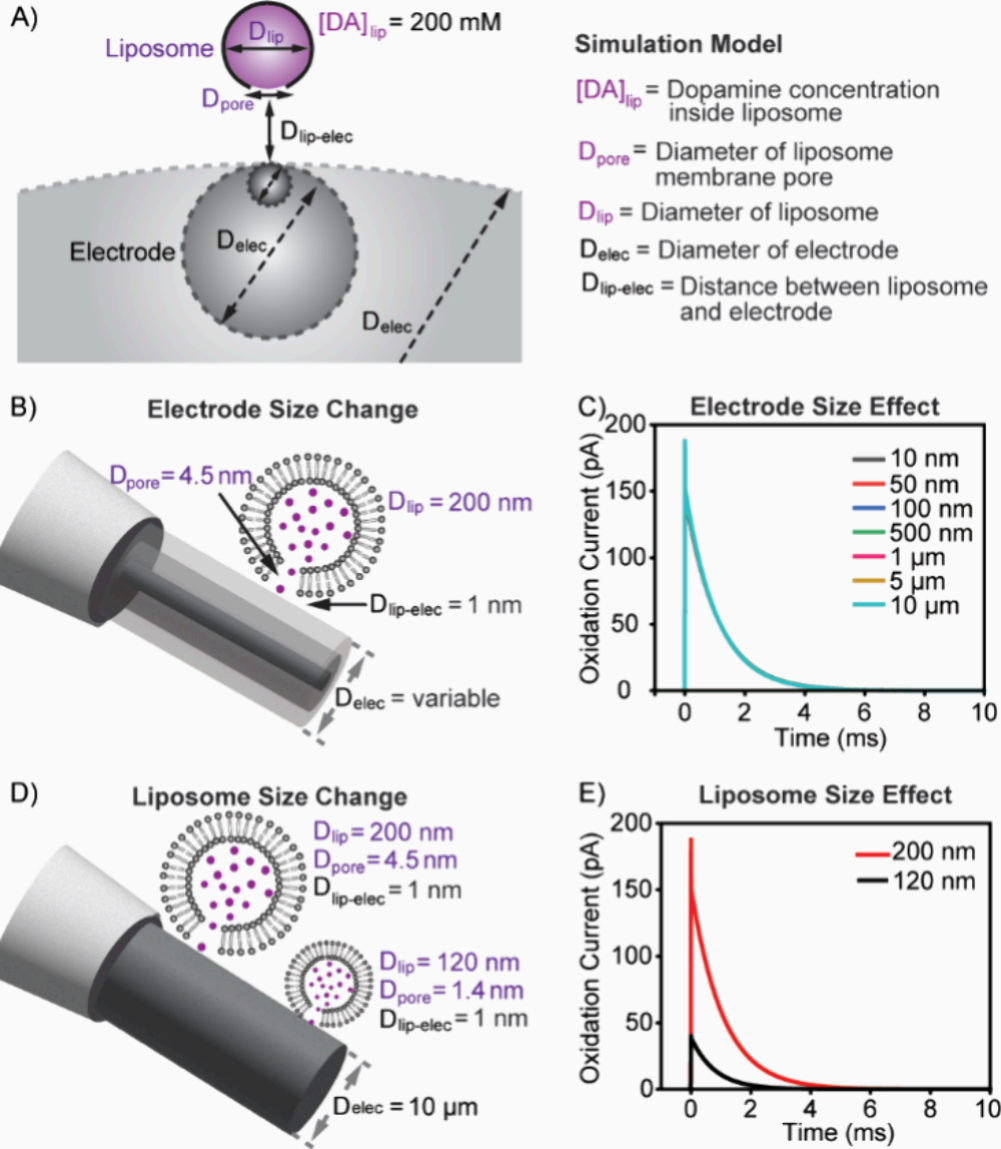
Prediction of amperometric DA detection efficiency as a function of electrode dimensions, liposome size, and spatial parameters relevant to the experimental amperometric recording of liposome rupture events. (A) Schematic of the simulation model used to evaluate DA detection efficiency by CFNEs from single liposomes, considering variation in electrode and liposome size, where the model computes DA release from an initial membrane pore, followed by the diffusion of DA through the pore, and subsequent diffusive flux to the electrode surface. (B) Illustration of the simulation framework used to assess how the CFNE diameter influences DA detection efficiency. (C) Simulated amperometric traces of DA release from a 4.5 nm membrane pore of a 200 nm liposome recorded using cylindrical electrodes with varying diameters (10 nm to 10 μm), showing how electrode size influences current spike shape and amplitude. Although seven conditions were simulated, the curves nearly completely overlap due to the minimal effect of electrode diameter under this configuration. (D) Schematic of the model used to evaluate the impact of liposome size on DA detection efficiency. (E) Simulated amperometric traces showing DA release from liposomes of two sizes, 200 nm (4.5 nm pore) and 120 nm (1.4 nm pore), interacting with a 10 μm cylindrical electrode, assuming a 1 nm liposome-electrode distance. All simulations assume a DA diffusion coefficient of 6 × 10^–10^ m^2^ s^–1^. Schematics are illustrative and are not drawn to scale.

We next varied the liposome-to-electrode distance (*d* = 5 and 20 nm) and electrode diameters, *D*_elec_ (10 nm to 10 μm) (Figure S4C,D). Although liposomes are expected to interact closely with the electrode surface before rupture, making a short *d* most probable scenario, simulations showed that minor diffusive losses at the smallest (*D*_elec_ = 10 nm); overall detection efficiency remained unaffected for the electrode sizes used in our experiments. Reducing *D*_pore_ to 3 nm similarly had no significant effect on the detection efficiency or peak shape across the electrode diameter range (Figure S4E). Thus, electrode geometry does not significantly influence mass transport or detection efficiency under our experimental conditions.

### Liposome Size as the Key Variable

Given the above, we considered an alternative explanation: differences in current spike characteristics may arise from variations in the size of liposomes that rupture at the electrode surface. In previous simulations, *D*_pore_ and liposome diameter were adjusted to match the experimental data from cone-shaped CFNEs. Since the amperometry method assumes complete liposome content release (in contrast to full or partial release in cellular exocytosis), a smaller measured *N* value indicates rupture of a smaller liposome. Comparing peak characteristics between the two different electrode types revealed that *N* was 81% smaller for needle-shape CFNEs (Table S1). To model this, we simulated DA release from 120 nm diameter liposomes. To reproduce similar *T*_1/2_ values for both electrodes, *D*_pore_ was reduced to 1.4 nm. Simulation performed at *d* = 1 nm and *D*_elec_ = 10 μm using 200 mM DA-loaded liposomes showed that current spikes from 120 nm liposomes closely matched experimental data (Figure 8D,E, Figure S4F). These results support the conclusion that CFNE geometry influences which subpopulation of liposomes ruptures at the electrode surface.

### Limitations of the Simulation Model

This model has several limitations. First, it does not account for the full size distribution of the liposomes. However, using average experimental parameters still provides a representative understanding of the system. Second, simulated peak currents exceeded experimentally measured values, possibly due to the incomplete release of DA from the liposomes. This does not invalidate the model as diffusion processes scale linearly with concentration. Lastly, the model assumes a static geometry. In reality, *D*_pore_ likely evolves dynamically during rupture. Still, by optimizing for the central peak parameters (*N* and *T*_1/2_), the model captures the key features of the amperometric event. Changes in *D*_pore_ would primarily influence the rise and fall of the peaks rather than the total charge.

### Biophysical Basis for Size-Selective Detection

Our findings support the hypothesis that CFNE geometry influences which liposomes are more likely to rupture based on size. This is consistent with known biophysical principles of lipid membranes, in particular, membrane curvature. While membranes generally resist stretching, they readily bend. Curving the membrane beyond the resting curvature stores elastic energy, which can drive conformational changes, such as fusion or fission.^27,28^ Membrane dynamics is closely influenced by the radius of membrane curvature, where both liposome shape and adhesion can alter membrane tension, usually in the range of a few mN/m, and can ultimately trigger membrane rupture.^29,30^

Smaller liposomes have higher curvature and smaller contact areas when adsorbed onto the electrode, which may reduce the extent of membrane deformation^31^ and limit exposure to the electrode’s electric field, where the electric field influences the formation of irreversible pores in the lipid bilayer, ultimately leading to liposome rupture.^32–35^ The high curvature of needle-shaped CFNEs may favor interactions with smaller liposomes by increasing the local mechanical deformation, thereby promoting rupture. In contrast, larger liposomes may not conform as readily to the needle-shape, reducing contact area and the probability of rupture.^36,31,37–39^ Cone-shaped CFNEs, having a lower curvature, may enable larger liposomes to adhere and spread more extensively, increasing the membrane area exposed to electric fields. This can elevate the membrane tension and rupture probability.

## Conclusions

This study introduces a robust, highly reproducible, and user-friendly method for fabricating short, well-defined carbon fiber CFNEs through stepwise electrochemical etching. The key innovation lies in combining real-time microscopy with micromanipulation to precisely position the CFME at the liquid-air interface of a KOH droplet, enabling the controlled application of single voltage pulses. This approach allows fine-tuned etching by adjusting potential pulse amplitude electrode positioning and etching duration, ultimately achieving precise control over CFNE shape and length while also preventing excessive etching that could compromise electrode functionality. The method reliably produces two CFNE geometries, cone-shaped and needle-shaped, with tip diameters of ∼100 nm and length limited to ∼10 μm, which is significantly shorter than those obtained with traditional methods, which typically range between 30 and 100 μm.^9^ Shorter electrodes eliminate the need for insulation of excess surface area as well as minimize background noise, reduce capacitive, and enhance sensitivity for amperometic detection.

Both CFNE types demonstrated excellent electrochemical performance and were used to record quantal DA release from individual DA-loaded liposomes. Importantly, analysis of these events, supported by diffusion-based modeling, revealed that electrode geometry influences liposome rupture behavior: highly curved needle-shaped CFNEs preferentially ruptured smaller liposomes, while broader cone-shaped CFNEs favored the rupture of larger ones. These findings underscore the critical role of nanoscale curvature in modulating membrane rupture and highlight the importance of electrode geometry in interpreting quantitative single-vesicle measurements.

While CFNE shapes do not affect detection efficiency once rupture occurs, they strongly influence which liposomes rupture, emphasizing the steric and biophysical interactions that govern electrode-liposome interfaces. Hence, understanding this interplay between electrode geometry and liposome size is essential for the quantitative interpretation of amperometric measurements using nanoelectrodes. This has important implications for studies involving heterogeneous vesicle populations, including secretory vesicles in biological systems.

In summary, this method offers a simple, cost-effective, and scalable strategy for producing CFNEs with a precisely tailored geometry and length, akin to nanoscale wood carving. By overcoming the limitations of existing fabrication techniques, it paves the way for more reproducible and electrochemical measurements in single-vesicle studies and other nanoscale analytical applications.

## Supplementary Material







## Notes

Additional data include evaluation of etching efficiency for nanotip electrode fabrication under various conditions (Figure S1), a comparison of electrochemical properties between nanotip electrodes and 30 μm disk electrodes (Figure S2), size distribution of 200 mM DA-loaded liposomes along with a summary of all liposome size measurements (Figure S3), *in silico* simulation of current spikes generated from oxidation of DA release from single DA-loaded liposome rupturing at the nanotip electrode surface (Figure S4), an illustration of the pulse generator used in this study (Figure S5), and a summary of kinetic details for single spikes burst measured by needle-shaped and cone-shaped nanotip electrodes (Table S1) (PDF)
Video 1: Fabrication of needle-shaped carbon fiber nanotip electrode (MPG)
Video 2: Fabrication of cone-shaped carbon fiber nanotip electrode (MPG)
