# Supplementary material for: Electrochemical Droplet Sculpturing of Short Carbon Fiber Nanotip Electrodes for Neurotransmitter Detection
Source: ACS Electrochem. 2025 Jun 16;1(9):1698–709. doi: 10.1021/acselectrochem.5c00135 (PMC12415824; doi:10.1021/acselectrochem.5c00135)
Supplement: Supplementary file 1 [file ec5c00135_si_001.pdf]

Supplementary Data

Electrochemical Droplet Sculpturing of Short Carbon Fiber Nanotip Electrodes for Neurotransmitter Detection

Yuanmo Wang[1], Pankaj Gupta[1], Ajay Pradhan[2], Raphael Trouillon[3,a,b], Jörg Hanrieder[2,4], Henrik Zetterberg[2,4,5,6,7,8], Ann-Sofie Cans[,1]*

[1] Department of Chemistry and Chemical Engineering, Chalmers University of Technology, Kemigården 4, Gothenburg 412 96, Sweden

[2] Department of Psychiatry and Neurochemistry, Institute of Neuroscience & Physiology, the Sahlgrenska Academy at the University of Gothenburg, Mölndal 431 80, Sweden

[3] Department of Electrical Engineering, Polytechnique Montréal, Montréal H3T 1J4, Canada

[a] TransMedTech Institute, Montréal H3T 1J4, Canada

[b] SNC Research Group, Montréal H3T 1J4, Canada

[4] Department of Neurodegenerative Disease, UCL Institute of Neurology, Queen Square, London WC1N 3BG, UK

[5] Clinical Neurochemistry Laboratory, Sahlgrenska University Hospital, Mölndal 431 80, Sweden

[6] UK Dementia Research Institute at UCL, London WC1N 3BG, UK

[7] Hong Kong Center for Neurodegenerative Diseases, Clear Water Bay, Hong Kong 999077, China

[8] Wisconsin Alzheimer's Disease Research Center, University of Wisconsin School of Medicine and Public Health, University of Wisconsin-Madison, Madison, Wisconsin 53726, United States

* Corresponding author: Ann-Sofie Cans (cans@chalmers.se)

Table of Content

- Etching Efficiency of Carbon Fiber Microelectrode (CFME) as a Function of Applied Voltage Amplitude and Voltage Pulse Duratio…….……………………………………3
- Comparison of Steady-State Current Amplitudes in Voltammograms of Carbon Fiber Nanotip Electrodes (CFNEs) and 30 µm Disk CFMEs………………………………..4
- Nanoparticle Tracking Analysis of Dopamine (DA)-Loaded Liposome Size …………5
- Kinetics and Dynamics of Individual Amperometric Current Spikes from DA Release During Rupture of DA-filled Liposomes Detected by CFNEs…………………………6

Simulated Amperometric Current Spikes for DA Detection from DA-filled Liposome Rupture at the CFNE Surface………………………………………………………...7

Etching Efficiency of Carbon Fiber Microelectrode (CFME) as a Function of Applied Voltage Amplitude and Voltage Pulse Duration.

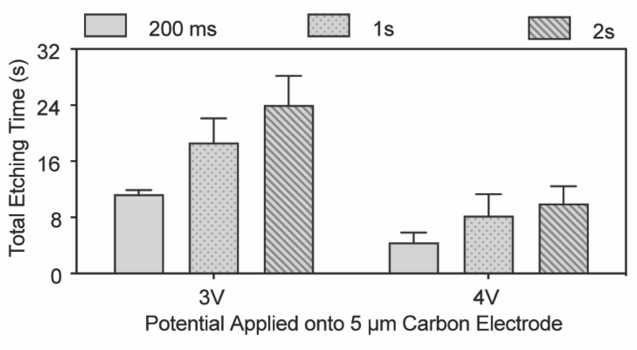

Figure S1. Etching efficiency assessed at varying voltage amplitudes (3V and 4V) and voltage pulse durations (200 ms, 1s and 2s). Data is presented as the mean ± standard error of the mean (SEM).

Comparison of Steady-State Current Amplitudes in Voltammograms of Carbon Fiber Nanotip Electrodes (CFNEs) and 30 µm Disk CFMEs.

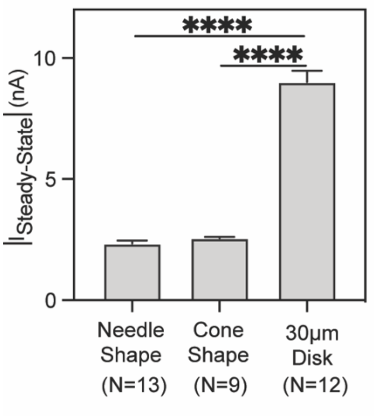

Figure S2. Steady-state current amplitude at 0.6 V was compared across three electrode types: needle-shaped CFNEs (n=13), cone-shaped CFNEs (n=9) and 30 µm disk CFMEs beveled at 45º angle (n=13). Voltage scans ranged from the - 0.1 V to 0.6V *vs* a Ag/AgCl reference electrode. The results are displayed as the mean ± SEM, and a Mann-Whitney (two-tailed unpaired) test was applied for statistical comparison ($^{****}p$ < 0.0001).

Nanoparticle Tracking Analysis of Dopamine (DA)-Loaded Liposome Size.

A)

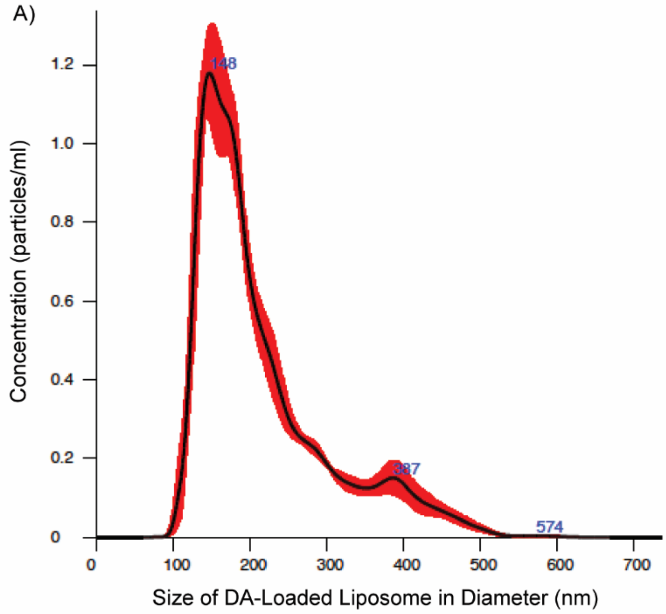

B) Size of DA-Loaded Liposome Measured by Nanoparticle Tracking Analysis.

	Measurement 1	Measurement 2	Measurement 3	Average ± SD*
Diameter in Mean (nm)	148	149	147	148 ± 1
Diameter in Mode (nm)	241	207	215	221 ± 18

*SD stands for standard deviation

Figure S3. (A) Representative frequency histogram showing the size distribution of synthesized liposomes filled with a 200 mM dopamine (DA) solution. (B) The measured and calculated average sizes of DA-loaded liposomes are reported as both the mean and mode, with the standard deviation included alongside the mean value.

Kinetics and Dynamics of Individual Amperometric Current Spikes from DA Release During Rupture of DA-filled Liposomes Detected by CFNEs.

Table S1. Summary of the kinetic and dynamic characteristics of individual amperometric spikes from 200 mM DA-loaded liposome bursts measured with needle-shaped (N = 4 measurements) and cone-shaped (N = 3 measurements) CFNEs.*

	T_{base} (ms)	$T_{1/2}$ (ms)	T_{rise} (ms)	T_{fall} (ms)	I_{max} (pA)	Q (fC)	Spike #
Needle Shape	1.00 ± 0.08	0.47 ± 0.04	0.23 ± 0.02	0.27 ± 0.02	6.43 ± 0.50	3.38 ± 0.52	707
Cone Shape	1.25 ± 0.02	0.55 ± 0.01	0.27 ± 0.01	0.27 ± 0.01	27.78 ± 1.50	17.52 ± 0.74	908

*The data presented is shown as the mean ± SEM.

Simulated Amperometric Current Spikes for DA Detection from DA-filled Liposome Rupture at the CFNE Surface.

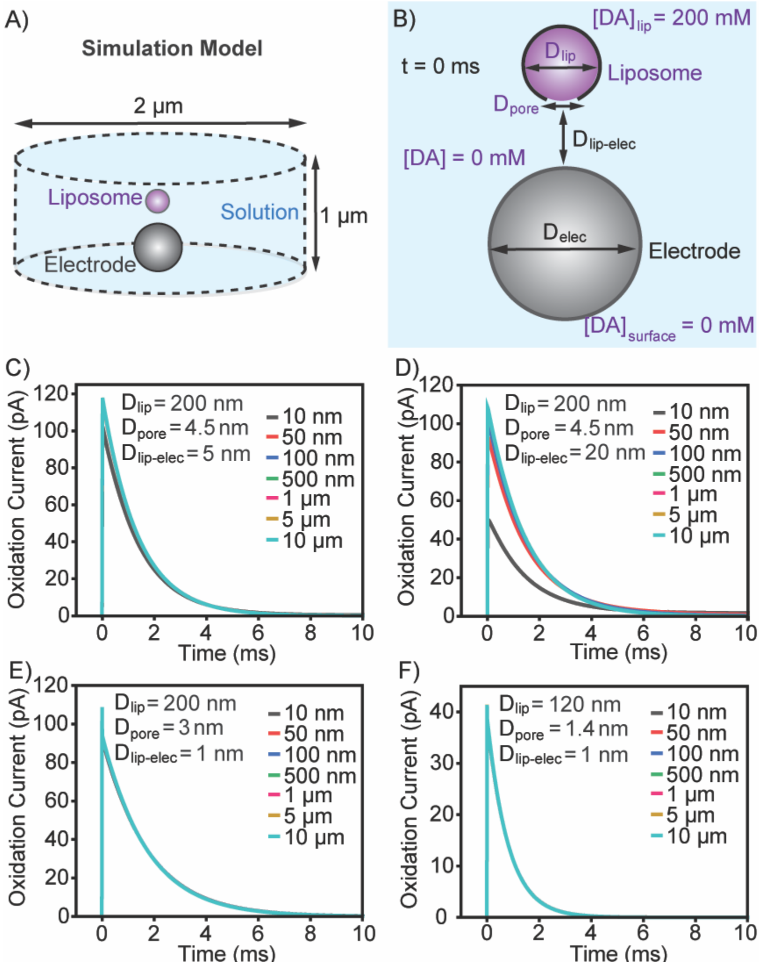

Figure S4. Prediction of amperometric current spikes generated by DA release from individual liposomes at the electrode surface. (A) Schematic of the simulation model: A buffer solution (blue cylinder) containing a single liposome (purple sphere) and an electrode (gray sphere) aligned along the vertical axis. (B) Initial state (t = 0 ms): A liposome with diameter D_{lip} is filled with 200 mM DA, surrounded by a DA-free buffer. The liposome membrane (black arc) is impermeable except for a membrane pore with diameter D_{pore}. The dopamine concentration [DA] at the electrode surface is initially set to 0 mM. (C, D) Predicted amperometric current spikes following rupture of a 200 nm liposome containing 200 mM DA at varying cylindrical electrode diameters (10 nm -

10 μm) and fixed membrane pore diameter of 4.5 nm. Liposome-electrode distances ($D_{lip\text{-}elec}$) are (C) 5 nm and (D) 20 nm. (E) Predicted amperometric current spikes for 200 nm liposomes with a 3 nm membrane pore, and (F) 120 nm liposomes with a 1.4 nm pore, both releasing 200 mM DA at varying electrode diameters (10 nm - 10 μm). In all simulation conditions (C–F), the current traces corresponding to different electrode diameters nearly completely overlap, indicating that under these conditions, electrode size has a negligible influence on the predicted amperometic response. All simulations were conducted assuming a diffusion coefficient for DA of 6 x 10^{-10} m^2 s^{-1}.
